# Relevance popularity: A term event model based feature selection scheme for text classification

**DOI:** 10.1371/journal.pone.0174341

**Published:** 2017-04-05

**Authors:** Guozhong Feng, Baiguo An, Fengqin Yang, Han Wang, Libiao Zhang

**Affiliations:** 1 Key Laboratory of Intelligent Information Processing of Jilin Universities, School of Computer Science and Information Technology, Northeast Normal University, Changchun, 130117, China; 2 Key Laboratory for Applied Statistics of MOE, Northeast Normal University, Changchun, 130024, China; 3 Institute of Computational Biology, Northeast Normal University, Changchun, 130117, China; 4 School of Statistics, Capital University of Economics and Business, Beijing, 100070, China; Tianjin University, CHINA

## Abstract

Feature selection is a practical approach for improving the performance of text classification methods by optimizing the feature subsets input to classifiers. In traditional feature selection methods such as information gain and chi-square, the number of documents that contain a particular term (i.e. the document frequency) is often used. However, the frequency of a given term appearing in each document has not been fully investigated, even though it is a promising feature to produce accurate classifications. In this paper, we propose a new feature selection scheme based on a term event Multinomial naive Bayes probabilistic model. According to the model assumptions, the matching score function, which is based on the prediction probability ratio, can be factorized. Finally, we derive a feature selection measurement for each term after replacing inner parameters by their estimators. On a benchmark English text datasets (20 Newsgroups) and a Chinese text dataset (MPH-20), our numerical experiment results obtained from using two widely used text classifiers (naive Bayes and support vector machine) demonstrate that our method outperformed the representative feature selection methods.

## Introduction

Text classification has been applied in many contexts, ranging from document indexing based on a controlled vocabulary, to document filtering, automated metadata generation, word sense disambiguation, hierarchical cataloguing of web resources, and in general any application requiring document organization or selective and adaptive document dispatching [1]. Many classification algorithms have been proposed for text classification, such as the naive Bayes (NB) classifier, k-nearest neighbors, and support vector machine (SVM) [2].

To classify documents, the first step is to represent the content of textual documents mathematically, after which, these documents can be recognized and classified by a computer. The vector space model is certainly employed, in which a document is represented as a vector in term space [3]. Because of the flexibility and complexity of natural language, the vocabulary expands rapidly as the amount of text increases. Vocabularies that are composed of tens of thousands of terms are very common in a nature corpus. Each dimension corresponds to a separate term, and dimensions of the learning space are called features in the general machine learning context. That is, each document is represented by a sparse and ultra-high dimensional vector, in which each element represents the term frequency within the document.

To reduce the dimension and improve classification performance, feature selection is the process of selecting features based on a training set. Representative feature selection methods such as Chi-square (CHI) and information gain (IG), which investigate the relationship between the class label of a document and the absence or presence of a term within the document based on statistical and information theory, have been proved to have a high-performance [4–7]. Recently, Bayesian feature selection methods are proposed in [8–10]. Qian and Shu [11] developed an efficient mutual information-based feature selection algorithm from incomplete data, which integrates the information theory and rough sets. Lin et al. [12] presented a novel framework with an optimization function to deal with multi-label feature selection with streaming labels. Zou et al. proposed a Max-Relevance-Max-Distance feature ranking method to find the optimized feature subset, which balances accuracy and stability of feature ranking and prediction task [13]. The method and software tool got good performance on several bioinformatics problems [14–16]. Zhou’s lab (Health Informatics Laboratory) described a feature selection algorithm, McTwo, to select features associated with phenotypes, independently of each other, and achieving high classification performance [17]. While, unsupervised methods select features when the document class labels are absente [18–20].

However, two features will be considered equally in a document by these methods even when they respectively have very different term frequencies (such as 1 and 10). As such, they will miss the importance of the more frequent terms within the document, and lead to the loss of information which may potentially enhance the feature selection performance.

Feature weighting is to measure feature’s contribution, which is another important process to improve classification performance for text classifiers such as SVM, kNN and so on. Term frequency information has gained much more attention in term weighing processes [21–25]. To accurately assign feature’s weight, Liu et al. in [26], proposed a novel constraint based weight evaluation using constrained data-pairs. These methods often contain a local weight factor and a global weight factor. Although the term frequency information within the documents is commonly employed in the local weighting factor, it rarely employed in the global weighting factor. Erenel and Altınçay confirmed that using term frequency in the global weight factor is beneficial for tasks which do not involve highly repeated terms [23].

Our motivation is to provide a good feature selection scheme by using the term frequency information within the documents in text classification. To this end, we investigated a widely used term event probabilistic model to capture term frequency information, borrowing from the ideal of relevance weighting [21, 27], and then get a novel feature selection measurement named *relevance popularity*. Finally, term frequency based intra-class association and term frequency based inter-class discrimination can be integrated naturally in our feature selection scheme.

The paper is organized as follows. The background of feature selection for text classification is given in Section 2. Section 3 describes the term event probabilistic model with NB assumption. In Section 4, we explain the newly proposed feature selection methods. Section 5 shows experiments and results. We conclude the paper with a brief discussion in Section 6.

## Related works

In this section, we will briefly describe some related works including the state-of-the-art feature selection methods used for text classification. To this end, we will introduce the bag-of-words model first. A toy example is given in Example 1.

**Example 1** *We have two documents*:

*d*_1_ *What do you do at work?*

*d*_2_ *I answer telephones and do some typing.*

Ignoring the term order, each document can be represent by a term frequency vector using the Bag-of-words model, namely, the number of times a term appears in the text [3]. For the example above, we can construct the following two lists to record the term frequencies of all the distinct words (Table 1):

**Table 1:** The term frequencies of all the distinct words.

	*t*_1_	*t*_2_	*t*_3_	*t*_4_	*t*_5_	*t*_6_	*t*_7_	*t*_8_	*t*_9_	*t*_10_	*t*_11_
	What	do	you	at	work	I	answer	telephones	and	some	typing
*d*_1_	1	2	1	1	1	0	0	0	0	0	0
*d*_2_	0	1	0	0	0	1	1	1	1	1	1

The number of features will increase rapidly as the number of documents increases, and many of them do not provide information for text classification. Feature selection is an essential step to improve the classification performance. Feature selection methods can be grouped into two main categories: document frequency (DF) based methods and term frequency (TF) based methods.

### DF based feature selection methods

Feature selection methods based on DF ignore the term frequency within each document, and instead use binary representation, (*B*_1_, *B*_2_, ⋯, *B*_*p*_), where *B*_*u*_ is a binary variable that indicates whether the document contains the term *t*_*u*_ or not. The label of the document can be denoted by *C*.

For simplicity and without loss of generality, we denote the feature (variable) *B*_*u*_ as *B*, and consider the 2-class classification problem. *N* is the number of documents in the training set, while some other notations are introduced in Table 2. Feature selection methods are often based on the number of documents, such as IG, CHI, the odds ratio, and so on.

**Table 2:** The numbers of the documents.

		Class	
		positive	negative
**Term**	occur	*a*	*c*
	not occur	*b*	*d*

IG is a synonym for Kullback–Leibler divergence in information theory and machine learning, which is used to measure the ability of a feature to distinguish the sample data. IG is given by

$$\begin{array}{cc} IG= & \frac{a}{N}\times \log\frac{a\times N}{\left(a+c)(a+b\right)}+\frac{b}{N}\times \log\frac{b\times N}{\left(b+d)(a+b\right)}\\ & +\frac{c}{N}\times \log\frac{c\times N}{\left(a+c)(c+d\right)}+\frac{d}{N}\times \log\frac{d\times N}{\left(b+d)(c+d\right)}. \end{array}$$ (1)

The CHI statistic is widely used in text classification as well as in other machine learning applications, which measures the independence between the random variable *B* and *C*, and is given by

$$CHI=N\times \frac{{\left(a\times d-b\times c\right)}^{2}}{\left(a+c)(b+d)(a+b)(c+d\right)}.$$ (2)

Li et al. proposed a supervised feature selection method, named CHIR, which is based on the *χ*^2^ statistic and new statistical data that can measure the positive term-category dependency [26].

These feature selection methods were proved to have a high-performance in text classification [4], although they do ignore the term frequency information within the documents.

### TF based feature selection methods

Recently, term frequency has gained more attention, not only in feature weighting [21, 23], but also in feature selection [28–30]. Among the TF based feature selection methods, Singh et al. defined a probabilistic popularity of a term by,

$$wcp_{u,k}=\frac{\mathrm{Pr}(t_{u}|C=k)}{\sum_{j=1}^{K}\mathrm{Pr}\left(t_{u}|C=j\right)},$$ (3)

where Pr(*t*_*u*_|*C* = *k*) is the conditional probability of term *t* given a class label *k* [31]. To analyze how a feature is distributed over different classes, they suggested to use the Gini coefficient of inequality to obtain the final feature selection measure, which they named the within class popularity (WCP).

After removing the normalize factor in Eq (3), only a term frequency based intra-class association factor is left. An additional inter-class discrimination factor may improve the performance of feature selection.

## Methods

Due to the good performance of WCP, we will revisit the probabilistic popularity of the terms and try to look for a model based scheme to measure the term information in this section.

### Term event model

**Fig 1:**
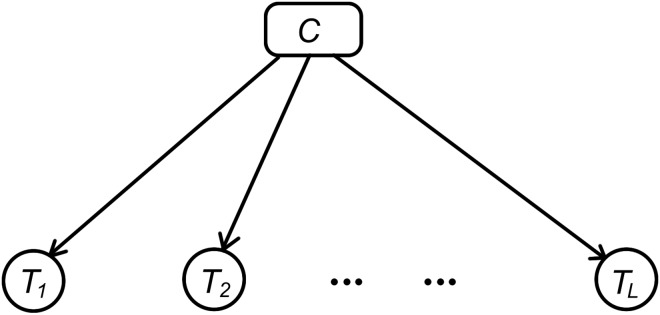
Graphic model representing the term event model with the NB assumption.

In statistical language modelling, a document is often regarded as a sequence of terms (words). The individual term occurrences are the “events” and the document is the collection of term events [32]. This model captures term frequency information in documents, and has been widely used for speech recognition and text classification. In mathematics, a document is represented by (*T*_1_, *T*_2_, ⋯, *T*_*L*_), where *L* is the length of the document. *T*_*l*_ is drawn from the vocabulary *V* = {*t*_1_, *t*_2_, ⋯, *t*_*p*_}, *l* = 1, 2, ⋯, *L*. In text classification, the order of events is often ignored. The NB assumption is that *T*_1_, *T*_2_, ⋯, *T*_*L*_ are independent given the document label variable, *C* [33], which can be illustrated by the graphic model in Fig 1.

Now we can obtain a *p*-dimensional vector ***X*** = (*X*_1_, *X*_2_, ⋯, *X*_*p*_) by

$$X_{u}=\sum_{l=1}^{L}I\left(T_{l}=t_{u}\right),\;\;u=1,2,\cdots ,p.$$

**Example 2** *Going back to* Example 1, *we have*

  *X*_1_  *X*_2_  *X*_3_  *X*_4_  *X*_5_  *X*_6_  *X*_7_  *X*_8_  *X*_9_  *X*_10_  *X*_11_

*d*_1_  *1* *2* *1* *1* *1* *0* *0* *0* *0* *0* *0*

*d*_2_  *0* *1* *0* *0* *0* *1* *1* *1* *1* *1* *1*

Then, for a document ***x*** = (*x*_1_, *x*_2_, ⋯, *x*_*p*_), the conditional probability function is

$$f\left(x|C=k\right)=\mathrm{Pr}\left(L\right)\times \frac{L!}{\prod_{u=1}^{p}x_{u}!}\prod_{u=1}^{p}{\mathrm{Pr}(t_{u}|C=k)}^{x_{u}},$$ (4)

where $L=\sum_{u=1}^{p}\;x_{u}$, Pr(*t*_*u*_|*C* = *k*) is the probability of {*T*_*l*_ = *t*_*u*_} in a document of class *k*.

#### Matching score functions

From the view of the Multinomial distribution in Eq (4), it is difficult to deal with the feature selection problem because of the internal dependencies among the features. In this section we will look for a new way, borrowing the matching score ideal from information retrieval [34, 35]. We first investigated the Multinomial NB classifier, and then derived a probabilistic feature selection scheme.

Without loss of generality, the binary text classification case was considered. Multi-class classification problems can be transformed into several two-class ones. For a new document, ***x*** = (*x*_1_, *x*_2_, ⋯, *x*_*p*_), and its class label, *C*, let *C* = 1 denote any document is from the positive class, and *C* = 0 for negative ones. Classification can be performed by calculating the posterior probability of the label given the document. By applying Bayes’ rule, we get

$$\mathrm{Pr}\left(C=1|x\right)=\frac{\mathrm{Pr}(C=1)\mathrm{Pr}(x|C=1)}{\mathrm{Pr}(x)}.$$

To avoid further expansion of Pr(***x***), we use the probability ratio rather than the probability. Thus, it satisfies the classification task:

$$\frac{\mathrm{Pr}(C=1|x)}{\mathrm{Pr}(C=0|x)}=\frac{\mathrm{Pr}(C=1)\mathrm{Pr}(x|C=1)}{\mathrm{Pr}(C=0)\mathrm{Pr}(x|C=0)}.$$

Ignoring the priori class probability ratio, the classification task can be achieved by the matching score function [35],

$$MS\left(x\right)=\log\frac{\mathrm{Pr}(x|C=1)}{\mathrm{Pr}(x|C=0)}=\sum_{u=1}^{p}x_{u}\log\frac{\mathrm{Pr}(t_{u}|C=1)}{\mathrm{Pr}(t_{u}|C=0)},$$ (5)

$$\sum_{u=1}^{p}\mathrm{Pr}\left(t_{u}|C=1\right)=1,\;\;\sum_{u=1}^{p}\mathrm{Pr}\left(t_{u}|C=0\right)=1.$$

The second equal sign in Eq (5) is established because of Eq (4). Hence, the matching score can be factorized into the local factors of each term.

#### Relevance popularity

Now, let us turn to the part of *x*_*u*_ in Eq (5). As *x*_*u*_ is the number of *t*_*u*_ in a new document, an appropriate substitute is the term occurrence probability to remove the influence of the document lenght. To describe the information provided by the term and identify the positive class documents, we define a matching score as

$$MS_{u}≜\mathrm{Pr}\left(t_{u}|C=1\right)\times \log\frac{\mathrm{Pr}(t_{u}|C=1)}{\mathrm{Pr}(t_{u}|C=0)}.$$ (6)

After replacing the probabilities by their Bayesian estimators based on the training data, we have a new measure *relevance popularity* (RP) as

$$rp_{u,1}=\frac{N_{u,1}+1}{N_{1}+p}\times \left|\log\frac{N_{u,1}+1}{N_{1}+p}-\log\frac{N_{u,0}+1}{N_{0}+p}\right|,$$ (7)

where *N*_*u*,1_, *N*_*u*,0_ are the term frequencies of *t*_*u*_ in the positive class and negative class, respectively. *N*_1_, *N*_2_ are the total term frequencies in the positive class and negative class, respectively. We used shrinkage estimators, known as Laplace smoothing, to allow the assignment of non-zero probabilities to terms which do not occur in the classes [36].

**Remark** RP has the following characteristics:

- The first part is the reigning part of WCP provided by Singh and Gonsalves [31]. A high value can represent a high association between a class and a term, i.e. the term occurs more frequently in documents of the class.
- The second part (in the absolute-value sign) can be regarded as an adjustment factor, and used to assign larger values to the discriminating terms.

**Fig 2:**
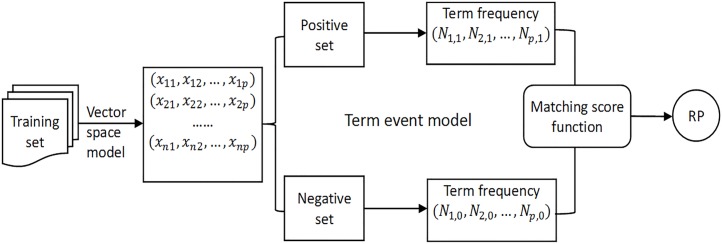
Block diagram of RP.

Hence, RP can not only capture informative terms, but also discriminating ones. A block diagram of our approach is shown in Fig 2, where our main idea may be summed up as follows: the larger popularity difference of a high-popularity term is between the positive category and the negative category, and the more contribution it makes when selecting the positive samples from the negative ones.

For a *K*-class classification problem, we first considered *K* two-class ones. For class *k*, we have

$$rp_{u,k}=\frac{N_{u,k}+1}{N_{k}+p}\times \left|\log\frac{N_{u,k}+1}{N_{k}+p}-\log\frac{N_{u,\tilde{k}}+1}{N_{\tilde{k}}+p}\right|,$$

where $N_{u,k},N_{u,\tilde{k}}$ are term frequencies of *t*_*u*_ in the positive class (i.e. class *k*) and the negative class (made up of the non-*k* classes), $N_{k},N_{\tilde{k}}$ are the total term frequencies, respectively.

**Example 3** *Back to* Example 1, *let* *d*_1_ *belong to class 1*, *and* *d*_2_ *belong to class 2*. *Consider the term* *t*_2_ *(i.e. “do”)*, *we can get*

*rp*_2,1_ = 0.0816, *rp*_2,2_ = 0.0514 *wcp*_2,1_ = 0.6136, *wcp*_2,2_ = 0.3864 after some calculation. However, widely used DF based feature selection methods CHI and IG cannot identify any difference.

#### Feature selection measure across the classes

Feature selection is to identify any features that discriminate between the classes. A good feature should have skewed information distribution across the classes. The Gini coefficient of inequality, which is a popular mechanism to estimate the distribution of income over a population, can be employed in our approach. After sorting *rp*_*u*,1_, *rp*_*u*,2_, ⋯, *rp*_*u*,*K*_ in increasing order, and denoting them by *rp*_*u*,(1)_, *rp*_*u*,(2)_, ⋯, *rp*_*u*,(*K*)_, we obtain the Gini coefficient estimator as

$$G\left(u\right)=\frac{\sum_{k=1}^{K}\left(2k-K-1\right)rp_{u,(k)}}{K\left(K-1\right){\bar{rp}}_{u}},$$ (8)

where ${\bar{rp}}_{u}=\frac{1}{K}\sum_{k=1}^{K}\;rp_{u,k}$ [31, 37].

## Experiments

In this study, we conducted two series of experiments under various experimental circumstances to evaluate the performance of the feature selection methods. To accomplish this, we compared three TF based feature selection methods (including our RP) and two DF based methods on a Chinese corpora and a popular benchmark data English corpora. We look for performance differences between the TF based feature selection methods and the DF based ones from the view of selecting features using the available Chinese dictionary in the first series of experiments. The second series experiments were performed to explore the superiority of the feature selection methods by the classification effectiveness using two state-of-the-art text classifiers: the Multinomial NB classifier and the SVM classifier.

### Feature selection methods

Feature selection methods, CHI and IG, were selected in our study due to their reported performance and typical representation in text classification [4]. To consider the term frequency information within the documents, the WCP [31] and T-test [30] methods were also included. Table 3 shows the summary of these methods.

**Table 3:** Summary of the feature selection Methods. CHI, IG are based on DF, and the others are based on TF.

CHI	measuring the dependence between a term and the document label
IG	the number of bits of information obtained for label prediction given a feature
RP	our newly proposed scheme based on term event model and the Gini coefficient
WCP	the Gini coefficient of within class probability
TT	the diversity of the distributions of a term between the specific class and the entire corpus, as based on the T-test

### Classifiers

Feature selection methods can be evaluated by further classification using the selected features. Two state-of-the-art text classifiers were chosen in our study, i.e. the Multinomial NB classifier and SVM. All algorithms were run using Matlab R2014b. For SVM, we employed LIBSVM-3.21, which is a integrated SVM software [38].

#### Multinomial NB

Multinomial NB is one of the most widely used and effective classifiers in text classification [33], which is based on the term event model. For a new document, ***x*** = (*x*_1_, *x*_2_, ⋯, *x*_*p*_), we have

$$\mathrm{Pr}\left(C=k|x\right)\propto \mathrm{Pr}\left(C=k\right)\times \prod_{u\in S}{\mathrm{Pr}(t_{u}|C=k)}^{x_{u}},\;\;\;\;k=1,2,\cdots ,K$$

where Pr(*C* = *k*) and Pr(*t*_*u*_|*C* = *k*) can be estimated based on the training data, S denotes the selected feature set. A document can be assigned a class label with maximum value of Pr(*C* = *k*|***x***). Hence, the effect of the feature selection schemes will have a direct bearing on the classification results. Feature selection methods can then be evaluated by the classification results.

#### Support vector machine

SVM is another method which is widely used and seems to have better performance than other methods in text classification. In our study, we adopt the linear SVM rather than the nonlinear SVM, as suggested in [21]. The reason is that the linear SVM is simple and fast and performs better than the nonlinear models.

### Text data collections

A Chinese text collection and a widely used English text collection were used in our experiment. The Chinese text collection was MPH-20, which is a subset of appeal call text records from the Mayor’s public hotline project in 2015 in the City of Changchun, China. After selecting the top 20 frequency functional departments (categories) and 1,000 documents from each class randomly, we obtained a MPH-20 text data set with 20,000 documents and 24,772 distinct terms, see S1 File. Table 4 shows the selected 20 categories of the appeal call text records.

**Table 4:** MPH-20: The categories of the appeal call text records.

Chaoyang District Government	Dehui Government	City Development and Reform Commission
Nanguan District Government	Jiutai District Government	Municipal Public Security Bureau
Kuancheng District Government	Nongan Government	Municipal Environmental Protection Bureau
Erdao District Government	Jingyue Development Zone	City Water Group
Shuangyang District Government	Economic Development Zone	Changchun Gas
Lvyuan District Government	Hi-tech Development Zone	City Transit Administration Bureau
Yushu Government	Automobile Development Zone	

The benchmark English collection was 20 Newsgroups (can be freely downloaded from http://qwone.com/∼jason/20Newsgroups/), which is a collection of approximate 20,000 news documents evenly divided among 20 groups. 18,774 total entries remained in this collection after removing duplicates, empty, single-word, and multi-labelled documents. 61,188 terms occurred in the corpus.

Table 5 shows some statistical information of those datasets, where *D* is the amount of documents, *p* is the size of the vocabulary, $\bar{L}$ is the average length of a document, *St*.*Dev* is the standard deviation of the document lengths, *D*_*train*_ is the size of the training set, and *D*_*test*_ is the size of the testing set.

**Table 5:** Statistical information of the two corpora.

Corpus	*D*	*p*	$\bar{L}$	*St*.*Dev*	*D*_*train*_	*D*_*test*_
MPH-20	20,000	24,772	43.46	32.51	10,095	9,905
20 Newsgroups	18,774	61,188	243.01	489.38	9,511	9,263

### Experimental results

#### Feature selection results

We use the available dictionary of MPH-20, and obtained the rank of terms using each feature selection method, see S2 File. Table 6 shows the top 20 Chinese terms selected by each method. From these results, TT (based on t-test) did not select new terms as compared with the results of IG and CHI. WCP found “driver”, “Jiutai” and “switch on”, which were not in the results of IG and CHI. Our proposed RP obtained quite different results, where all of the top 20 terms were not selected by the comparing methods. From the selected terms, we can see RP selected terms with detailed meaning and high frequency within the documents, such as “take an exam”, “chauffeured car”, “Boshuo road” and so on. Any terms that often occurred no more than once within the documents were not included in the top 20 terms, such as “Yushu city”, “Shuangyang district”, “gas”, “citizen” and so on.

**Table 6:** MPH-20: Top 20 Chinese terms using each feature selection method.

RP	WCP	TT	IG	CHI
Take an exam	Yushu city	Shuangyang district	Shuangyang district	Shuangyang district
Chauffeured car	Shuangyang district	Yushu city	Yushu city	Nongan county
Boshuo road	Dehui city	Nongan county	Nongan county	Yushu city
Heilin town	Jiutai city	Dehui city	Dehui city	Dehui city
Daqing	Nongan county	Jingyue development zone	Erdao district	Jiutai city
Shuangde township	Gas corporation	Automobile development zone	Kuancheng district	Automobile development zone
Suitcase	Automobile development zone	Nanguan district	Nanguan district	Jingyue development zone
Operate	Gas	Chaoyang district	Chaoyang district	Gas
Yunshan	Jingyue development zone	Erdao district	Jingyue development zone	Erdao district
Cremation	High-tech development zone	Jiutai city	Lvyuan district	Economic development zone
Wanjinta township	Economic development zone	Kuancheng district	Automobile development zone	High-tech development Zone
Kaoshan town	Water group	Lvyuan district	Jiutai city	Lvyuan district
Gongpeng town	Driver	Economic development zone	Economic development zone	Nanguan district
Gong	Erdao district	High-tech development zone	Gas	Kuancheng district
Yuxi street	Taxi	Village	High-tech development zone	Chaoyang district
Rename	Jiutai	Gas	Villager	Gas corporation
Longjia town	Switch on	Villager	Citizen	Water group
Shanghewan	Chaoyang district	Citizen	Village	Water pause
Gaming machine	Nanguan district	Water pause	Water pause	Charge
Festival	Lvyuan district	Water group	Gas corporation	Taxi

#### Classification performance results

**Fig 3:**
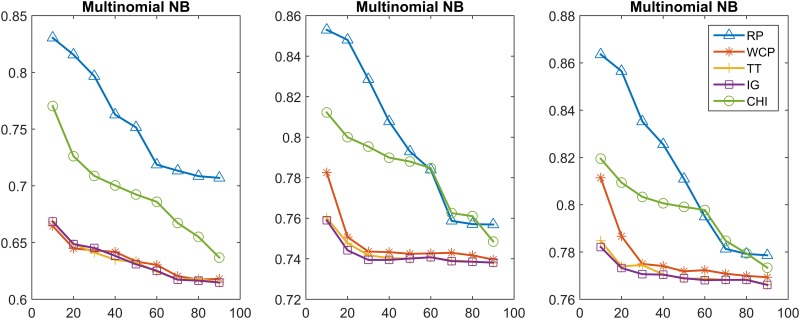
MPH-20: The classification accuracy values of five feature selection methods when using the Multinomial NB classifier.

**Fig 4:**
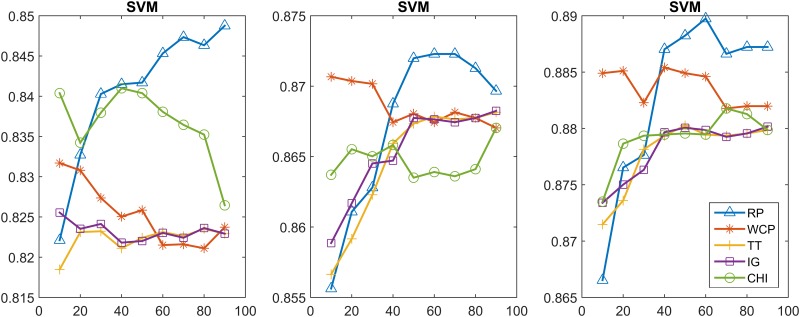
MPH-20: The classification accuracy values of the five feature selection methods when using the SVM classifier.

**Fig 5:**
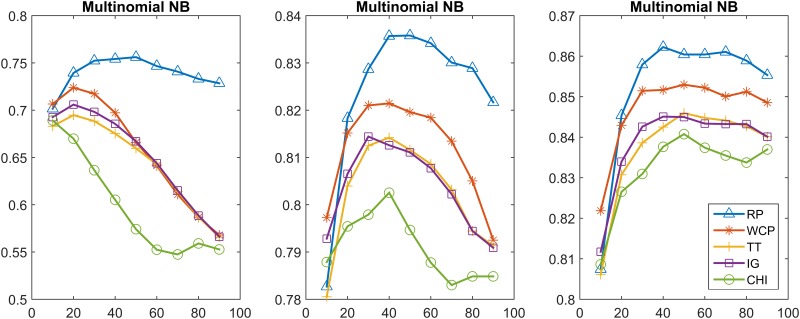
20 Newsgroups: The classification accuracy values of the five feature selection methods when using the Multinomial NB classifier.

**Fig 6:**
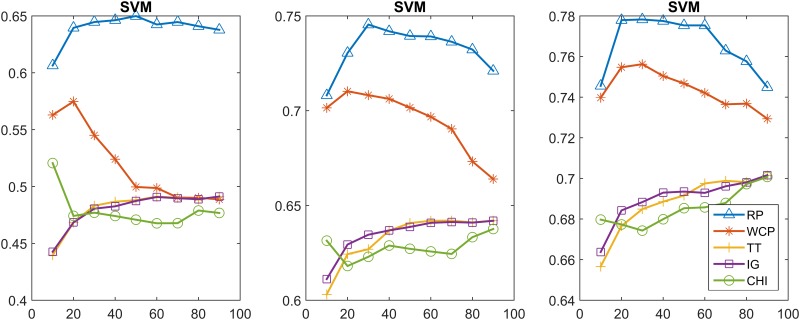
20 Newsgroups: The classification accuracy values of the five feature selection methods when using the SVM classifier.

In this section, we further compare the performance of the feature selection methods using the Multinomial NB and linear SVM classifiers. In particular, we achieved the classification model by incremental training using 20%, 60%, 100% of the training set. Figs 3–6 show the classification results obtained from using the Multinomial NB and SVM text classifiers on the MPH-20 and 20 Newsgroups datasets. 20%, 60%, 100% of the training set were used from the left to the right. Each curve of these figures represents a different feature selection method.

Fig 3 depicts the classification accuracy performance of five different feature selection methods (i.e., RP, WCP, TT, IG, CHI) on MPH-20 when using the Multinomial NB text classifier. All methods obtained their best values when 10% of the features were included. RP outperformed all the contrast methods, with the best accuracy value 0.8636, whereas WCP, TT, IG, CHI obtained 0.8114,0.7848,0.7821,0.8195, respectively, when the entire training set was used. All of the methods obtained better results when the size of training set increased. In all training cases, there were downtrends when more features were included.

Fig 4 depicts the classification accuracy performance using SVM. The performance trends of the different feature selection methods are different. For RP, the accuracy reach a peak (0.8723 and 0.8898) at a feature size of 60%, when either 60% or 100% of the training set was used. In the case of using the 20% training set, the RP accuracy showed a tendency to increase as the number of features grew, and obtained a best value of 0.8488 when using the whole training set. WCP, TT, IG, CHI obtained a best accuracy of 0.8854,0.8803,0.8802,0.8818, respectively.

Fig 5 depicts the classification accuracy performance on 20 Newsgroups when using the Multinomial NB. The trends of the different curves are similar for each case. All of the methods obtained better results when the size of the training set increased. RP outperformed all of the contrast methods with a best accuracy vaoflue 0.8622, where WCP, TT, IG, CHI obtained 0.8522,0.8459,0.8451,0.8408, respectively.

Fig 6 depicts the classification accuracy performance when using SVM. All of the methods obtained better results when the size of the training set increased. Furthermore, the performance trends of the different feature selection methods were similar in all cases this time. The RP accuracy reached a peak value of 0.7783 when using the entire training set. WCP, TT, IG, CHI obtained a best accuracy of 0.7562,0.7014,0.7016,0.7007, respectively.

#### Feature selection number determination

In this section, we will determine the feature selection number. We suggest to use cross-validation to choose the best feature selection percentage on the training set. For each method, we employed 5-fold cross-validation and tried the following percentages in our experiment: 10%,20%,30%,40%,50%,60%,70%,80%,90%.

Table 7 shows the classification accuracy values and the including feature numbers of the feature selection methods on MPH-20. When using the Multinomial NB classifier, RP got the best accuracy 0.8636. CHI got the second best accuracy 0.8195, which is much smaller. WCP got 0.8114, TT and IG performed less well. All methods selected 1,915 features. When using the SVM classifier, RP got the best accuracy 0.8872 and included 17,242 features. WCP got the second best accuracy 0.8851 and included 3,831 features. CHI got 0.8818, TT and IG performed less well. All methods selected more than 13,000 features except WCP.

**Table 7:** MPH-20: The classification accuracy values (*A*) and the including feature numbers *N* of the five feature selection methods. The largest accuracy value and the smallest feature numbers are highlighted in bold for each classifier.

	RP		WCP		TT		IG		CHI	
Classifier	*A*	*N*	*A*	*N*	*A*	*N*	*A*	*N*	*A*	*N*
Multinomial NB	**0.8636**	**1,915**	0.8114	**1,915**	0.7848	**1,915**	0.7821	**1,915**	0.8195	**1,915**
SVM	**0.8872**	17,242	0.8851	**3,831**	0.8794	13,410	0.8796	15,326	0.8818	13,410

Table 8 shows the classification accuracy values and the including feature numbers of the feature selection methods on 20 Newsgroups. When using the Multinomial NB classifier, RP got the best accuracy 0.8604 and included 32,326 features. WCP got the second best accuracy 0.8517 and included 21,550 features. TT got 0.8459 and included 26,938 features. IG and CHI performed less well. When using the SVM classifier, RP got the best accuracy 0.7753 and included 26,938 features. WCP got the second best accuracy 0.7547 and included 10,775 features. TT, IG and CHI performed less well. RP and WCP selected much less features than other methods.

**Table 8:** 20 Newsgroups: The classification accuracy values (*A*) and the including feature numbers *N* of the five feature selection methods. The largest accuracy value and the smallest feature numbers are highlighted in bold for each classifier.

	RP		WCP		TT		IG		CHI	
Classifier	*A*	*N*	*A*	*N*	*A*	*N*	*A*	*N*	*A*	*N*
Multinomial NB	**0.8604**	32,326	0.8517	**21,550**	0.8459	26,938	0.8451	**21,550**	0.8376	**21,550**
SVM	**0.7753**	26,938	0.7547	**10,775**	0.7014	48,489	0.7016	48,489	0.7007	48,489

#### Discussion

The feature selection results of the TF based methods (RP, WCP and TT) and two DF based methods (IG and CHI) on MPH-20 demonstrate that our method has the advantage of using the term frequency select the terms with more details and important (high frequency within the documents) information.

Furthermore, the classification results when using both the NB and SVM classifiers and different training set sizes on the MPH-20 and 20 Newsgroups datasets illustrate the superiority of RP compared with the state-of-the-art feature selection methods.

## Conclusions and future work

We proposed a novel feature selection scheme via a widely used probabilistic text classification model. We captured term frequency information within the documents via a term event Multinomial model. To remove complex factors, we employed the logarithmic ratio of the positive class posterior probability to the negative one (e.g. the matching score idea). Then, we obtained a sub-score named *relevance popularity* of each feature under the well known NB assumption. Finally, we obtained a global feature selection score by using the Gini coefficient estimator [31, 37].

Experiments on the MPH-20 and 20 Newsgroups datasets that used both NB and SVM classifiers verified that the proposed feature selection scheme has the advantage of the term event model, which provides better scores than exiting methods for text classification problems.

The proposed *relevance popularity* coupled with the Gini coefficient has an appreciable advantage for text classification problems. Future works may consider the optimal choice of the global goodness function for relevance popularity and obtain some theoretical results.

## Supporting information

S1 File MPH-20 Data. The Chinese text data set used in the experiment.
(MAT)

S2 File Feature Selection Results. The feature selection score ranks on MPH-20.
(XLSX)
